# Lynch-like syndrome with germline WRN mutation in Bulgarian patient with synchronous endometrial and ovarian cancer

**DOI:** 10.1186/s13053-023-00257-1

**Published:** 2023-07-14

**Authors:** Zornitsa Bogomilova Kamburova, Polina Damyanova Dimitrova, Diana Strateva Dimitrova, Katya Stefanova Kovacheva, Savelina Lubenova Popovska, Slavena Enkova Nikolova

**Affiliations:** 1 Department of Medical Genetics, Medical University – Pleven, Pleven, Bulgaria; 2 Center of Medical genetics, University Hospital “Dr. Georgi Stranski”, Pleven, Bulgaria; 3 Department of Pathoanatomy, Medical University – Pleven, University Hospital “Dr. Georgi Stranski” – Pleven, Pleven, Bulgaria; 4 Clinic of Oncogynecology, University Hospital “Dr. Georgi Stranski” – Pleven, Pleven, Bulgaria

**Keywords:** Lynch-like syndrome, MMR deficiency, Proband, Likely pathogenic variant, WRN gene, Genetic counseling

## Abstract

**Background:**

Synchronous endometrial and ovarian cancer (SEOC) accounts for 50–70% of all synchronous gynecology cancers in women. Approximately 14% of SEOC cases are caused by Lynch syndrome (LS). The widespread introduction of “universal screening” at LS (all cases with CRC and all EC cases diagnosed before age 60 should be tested for MMR deficiency) has led to an increasing number of suspected LS cases- MMR-deficient tumors without germline mutation in the MMR genes. These cases are attributed to the so-called Lynch-like syndrome (LLS).

**Case presentation:**

We present a case of LLS with a detected germline, likely pathogenic variant in the WRN gene. The proband was a woman diagnosed with SEOC at the age of 51 years. Histology of both tumors (endometrium and ovary) was endometroid and showed loss of MLH1 and PMS protein expression. Genetic testing by next generation sequencing (NGS) detected a germline mutation (in the heterozygous state) in the WRN gene - c.4109del, p.(Asn1370ThrfsTer23) in the proband.

**Conclusions:**

The presented case contributes to the etiology of LLS and confirms the need for specific genetic testing, together with genetic counseling, in hereditary cancer syndromes. The use of combined information from clinicians, pathologists, genetic counselors, and data from NGS testing for cancer predisposition, clinical surveillance, and follow-up management in women with gynecology cancers, especially SEOC, could be improved.

## Background

The simultaneous occurrence of endometrial and ovarian cancer, known as synchronous cancer (SEOC), occurs in approximately 5% of cases of endometrial cancer and 10% of cases of ovarian carcinoma [1], and it is estimated that up to 14% are caused by Lynch syndrome [2, 3].

Lynch syndrome (LS) is an autosomal dominant cancer syndrome characterized by a high risk of predominantly colorectal and endometrial cancer (lifetime risk of up to 60%), but also ovarian, pancreatobiliary, urinary tract, brain, and sebaceous gland cancers [4, 5]. It is caused by germline mutation in one of the DNA mismatch repair (MMR) genes - *MLH1* (3p22), *MSH2* (2p21), *MSH6* (2p16), *PMS2* (7p22), *MLH3* (14q24), *MLH2* (2q32) [6]. According to Knudson’s hypothesis, the pathogenesis of hereditary cancers, particularly LS, starts with a first hit - an inherited germline mutation in tumor suppressor genes (MMR genes). The second hit is somatic mutation, leads to inactivation of the MMR mechanism, resulting in accumulation of numerous mutations, most evident in repetitive DNA during replication [7]. This tumorigenesis in LS causes the following characteristics of tumors - microsatellite instability (MSI), loss of MMR proteins, and a high number of somatic mutations, all of listed above together are referred to as MMR deficiency [8, 9]. MMR deficiency can also occur in some sporadic cases of colorectal (CRC) and endometrial cancer (EC), and the most common cause is hypermethylation of the *MLH1* promoter, leading to loss of expression of the *MLH1* and *PMS2* proteins [10].

The differentiation of tumors into LS and sporadic cases is very important for the follow-up of patients with LS and for their relatives [11]. More recently, the widespread introduction of “universal screening” for LS (all cases with CRC and all EC cases diagnosed before age 60 should be tested for MMR deficiency) [12, 13] has led to an increasing number of suspected LS cases- MMR-deficient tumors without germline mutation in the MMR genes. These cases are attributed to the so-called Lynch-like syndrome (LLS) [14]. LLS tumors have been shown to account for up to 70% of patients with MSI and MMR suspected for LS [15, 16]. The prevalence of LLS is 56–71% in CRC and between 30 and 64% in EC [17]. Thus, the prevalence of cases with LLS is approximately twice as high in both CRC and EC as in LS.

The aetiology of LLS is not yet clear, but three possible mechanisms are suspected: (a) germline mutation in other genes involved in MMR that could also cause MMR deficiency in tumor tissue, (b) germline mutation in MMR genes that cannot be identified due to the insufficiency of the performed DNA test, (c) somatic mutations within tumor cells causing the same MMR deficiency. Therefore, LLS patients are a heterogeneous group that includes sporadic cases with biallelic MMR deficiency and inherited cases related to pathogenic germline variants in other DNA repair genes [18, 19]. LLS cases cannot be easily attributed to inherited or sporadic MMR deficiency, which complicates the management of patients. Carriers of hereditary MMR deficiency and their carrier relatives are at high risk for a second primary carcinoma or for developing cancer and they should be referred for prevention. In contrast, individuals with somatic inactivation of MMR and their relatives are not at such increased risk. While the risk of cancer in cases with LS is generally higher than in general population (particularly for CRC and EC) and there are guidelines for the treatment and surveillance for these patients and their relatives [20, 21], the risk of cancer associated with LLS is unclear. Different studies show conflicting results regarding age of diagnosis, risk of CRC and EC [19].

The genetic basis of hereditary LLS is not yet fully understood. With the advent of next generation sequencing (NGS) and the ability to test many cancer-predisposing genes simultaneously, many have been shown to cause inherited cases of LLS - *MUTYH* [22], genes involved in cell activity regulation (*EXO1, POLD1, RCF1*, and *RPA1*) [23], *BUB1* and *BUB3* [24], *SETD2* [24], *WRN* [25], *BARD1* [25], and other genes that promote genomic integrity [25].

We present a case of LLS with synchronous endometrial and ovarian cancer and detected germline pathogenic variant in the *WRN* gene.

### Patient

The index patient (proband) was referred to the Center of Medical genetics in University Hospital “Dr. Georgi Stranski” – Pleven for germline genetic testing. Blood sample was obtained (in EDTA tube) from the patient after informed consents.

### IHC procedure

Tumor sample used in the present study was collected after obtaining informed consent for participation in the study. Endometrial tumor specimens from the proband was fixed in 10% buffered formalin for 24–36 h at room temperature, dissected and paraffin embedded. A pathologist selected 5 μm thick parallel sections of representative invasive tumor material and normal mucosa, and the tissue sample was confirmed to contain cancerous tissue using hemoxylin and eosin staining, which was performed as routine. Epitope retrieval time for all tumor sections was 20 min at 97˚C in DAKO PT Link (cat. no. PT100/PT101). Tumor sections were stained with the following antibodies (all from Dako, Agilent Technologies, Inc., and all came ready to use): ES05Monoclonal mouse AntiHuman MutL Protein Homolog 1, (cat. no. IR079), FE11Monoclonal mouse AntiHuman MutS Protein Homolog 2 (cat. no. IR085), EP49Monoclonal rabbit AntiHuman MutS Protein Homolog 6 (cat. no. IR086), and EP51Monoclonal rabbit AntiHuman Postmeiotic Segregation Increased 2 (cat. no. IR087) for MLH1, MSH2, MSH6 and PMS2 respectively. Incubation time for all antibodies was 20 min at room temperature. Staining was done with Autostainer Link 48 (Dako Agilent) slide stainer was used according to the manufacturer’s protocol. The external negative controls were the negative reagent controls included in the kit.For internal positive controls were used normal endometrial mucosa, stromal cells and stromal lymphocytes from the same patients. Results were analyzed manually by a pathologist. Expression was reported as: Normal, (retained expression) nuclear expression in > 10% tumor cells and retained expression in the internal control or Negative, (loss of expression) 0% expression in tumor cells and retained expression in the internal control.

### Germline pathogenic variant detection

Genomic DNA was isolated from blood sample using MagCore Genomic DNA Whole blood Kit according to the manifacturer’s protocol.

The genetic testing of the proband was performed by next generation sequencing (NGS). Trusight Cancer Sequencing Panel (Illumina©) was used for library preparation. The pan-hereditary cancer panel contained oligo probes targeting 94 genes and 284 SNPs associated with increased cancer predisposition. The procedures following the manufacturer’s instructions. Qualified libraries were sequenced on the Illumiina NextSeq 550 platform with 2 × 150 bp configuration. Reads were aligned to the reference human genome hg19. Data output files (gVCF) were imported into BaseSpace Variant Interpreter (Illumina©). Custom filters (included a minimum read depth of 20x per variant and excluded silent variants) were created to improve variant annotation and interpretation. The five-tier terminology system of the American College of Medical Genetics and Genomics (ACMG) was used for variant classification [26], including: Pathogenic (P), Likely Pathogenic (LP), Variant of Unknown clinical significance (VUS), Likely Benign (LB), and Benign (B). The variants automatically annotated by the software were manually checked in the main human genome databases: ClinVar (www.ncbi.nlm.noh.gov/clinvar), dbSNP (www.ncbi.nlm.noh.gov/projrct/SNP), and Ensembl (http://www.ensembl.org).

## Results

The proband was a 51-year-old women, nulliparous, postmenopausal (menopause at 49 years), underwent abrasion probatoria, because of vaginal bleeding. Histopathological examination of biopsy specimens indicated endometrial adenocarcinoma (highly differentiated, endometroid). The patient underwent second surgery under general anesthesia - total abdominal hysterectomy and bilateral salpingo-oophorectomy.

**Fig. 1:**
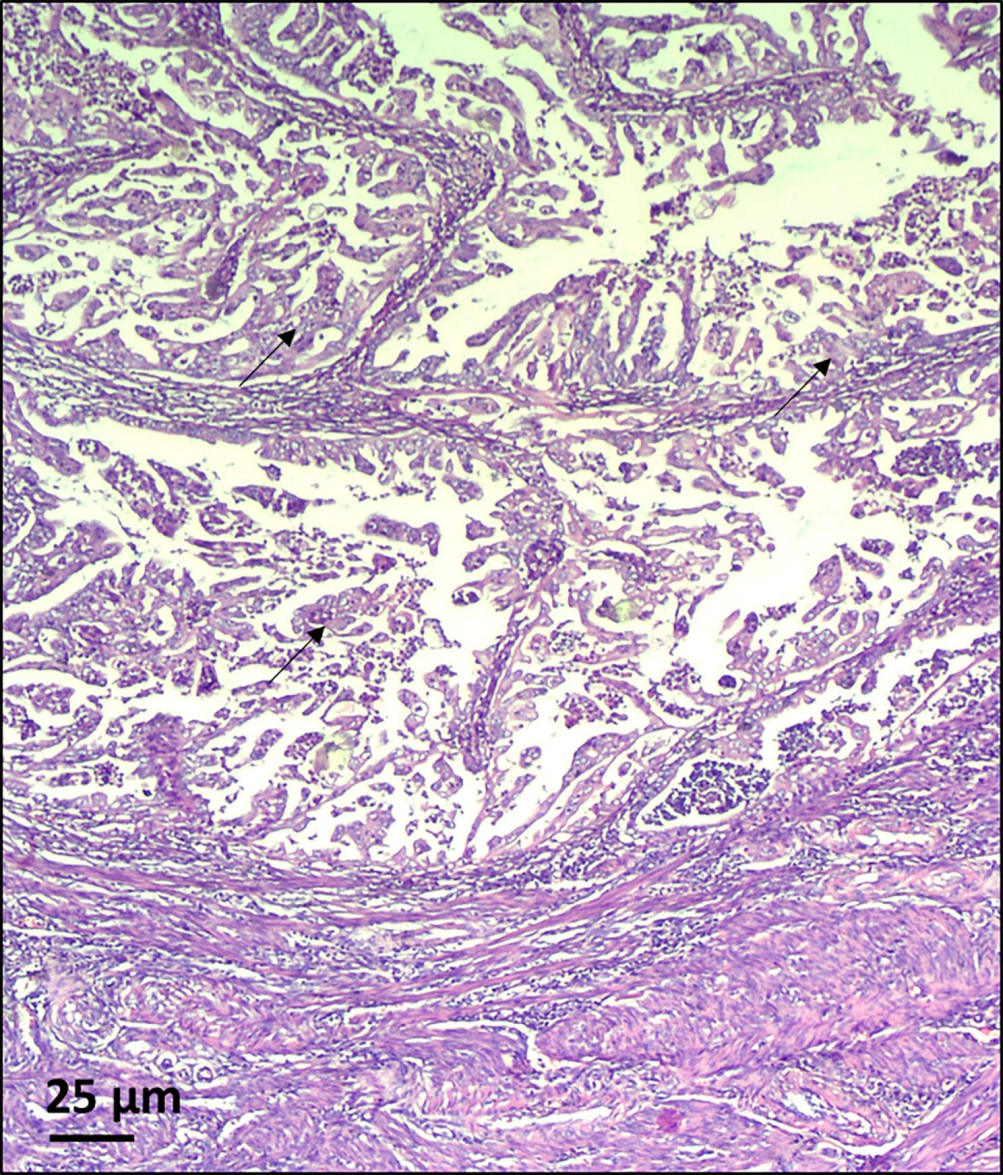
Endometrial adenocarcinoma (black arrow). The material obtained from the proband showed a highly differentiated endometroid endometrial adenocarcinoma. Samples were analyzed using hematoxylin and eosin staining. Magnification x100

**Fig. 2:**
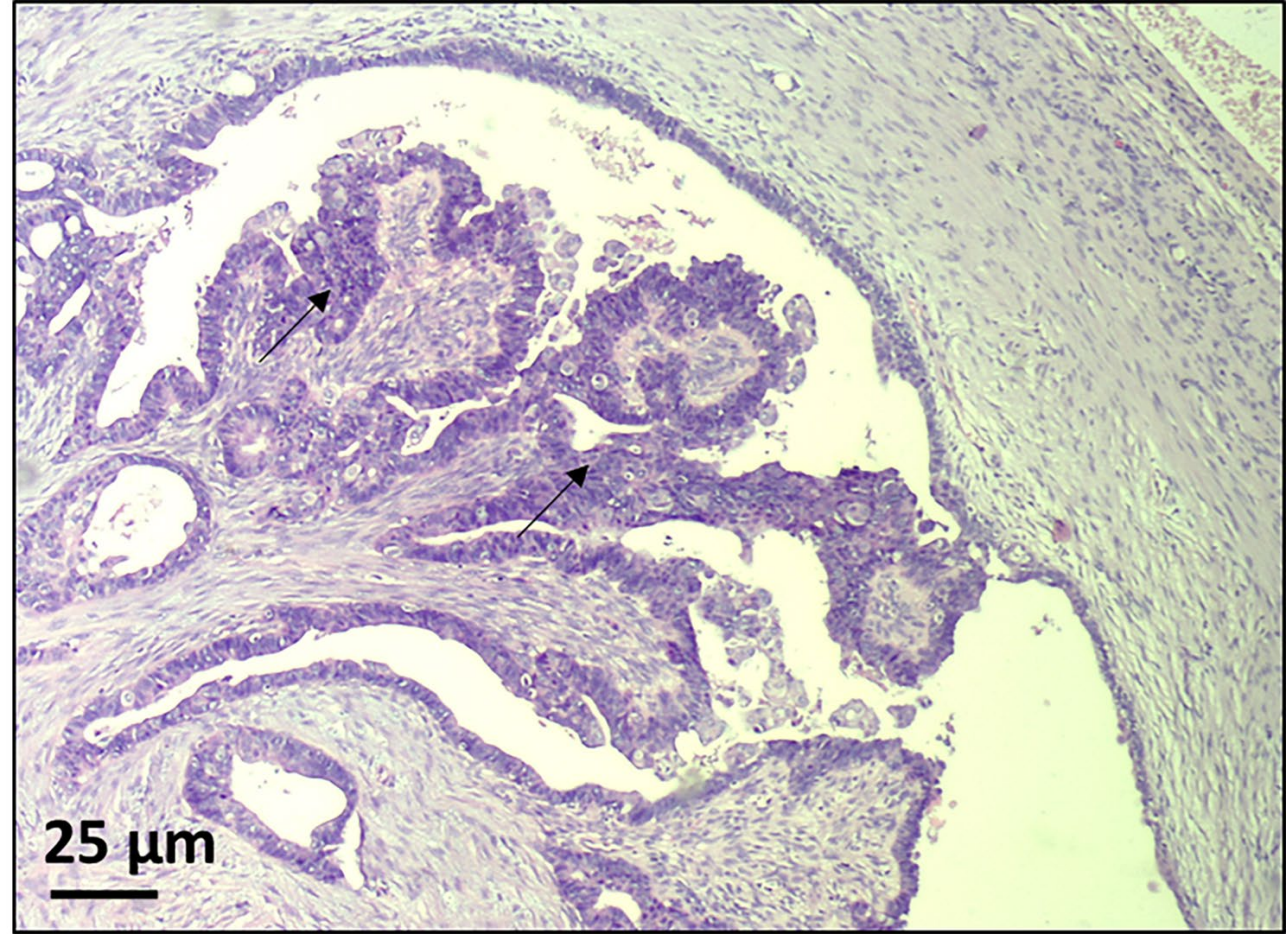
Ovarian cancer (black arrow). The material obtained from the proband showed an endometroid ovarian cancer. Samples were analyzed using hematoxylin and eosin staining. Magnification x100

The histopathological diagnosis from the second surgery revealed synchronous cancers (highly differentiated endometroid endometrial adenocarcinoma (Fig. 1) and endometroid ovarian cancer (Fig. 2) as well as lymph node with a metastasis. The therapy was pelvic radiotherapy, followed by four courses of platinum-based chemotherapy.

**Fig. 3:**
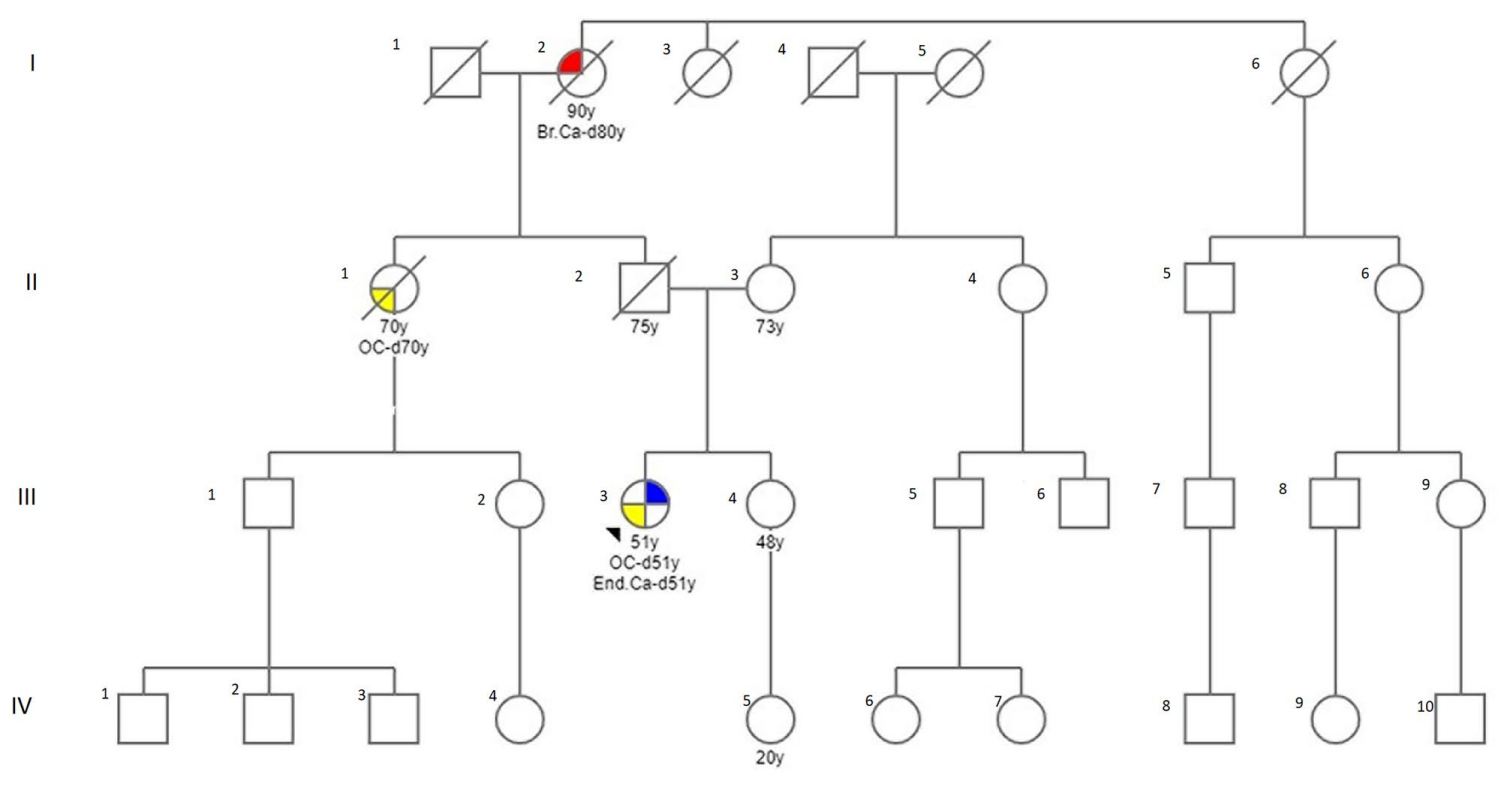
Genealogy of the family. Analysis included 31 individuals from four generations (numbers of generations are indicated with Roman numerals); tree of the family members were affected by a cancer: End. Ca, endometrial cancer (symbols with filled right upper quadrant); OC, ovarian cancer (symbols with filled left lower quadrant), Br.Ca.- breast cancer (symbols with filled left upper quadrant). The proband is indicated by an arrow. Circles are females, squares are males, diagonal slash indicates a deceased individual, the current age/age at death of individuals and the age at diagnosis (indicated with d.) are below the symbols

The patient was advised to undergo germline genetic testing for an inherited predisposition to cancer and she came to our Center of Medical Genetics for testing. The parents of the proband were healthy and father died of a heart attack at age 75. Genealogy revealed two relatives (II degree) with cancer - one with ovarian cancer (diagnosed at age 69 years) and the other with breast cancer (diagnosed at age 80 years) (Fig. 3).

**Fig. 4:**
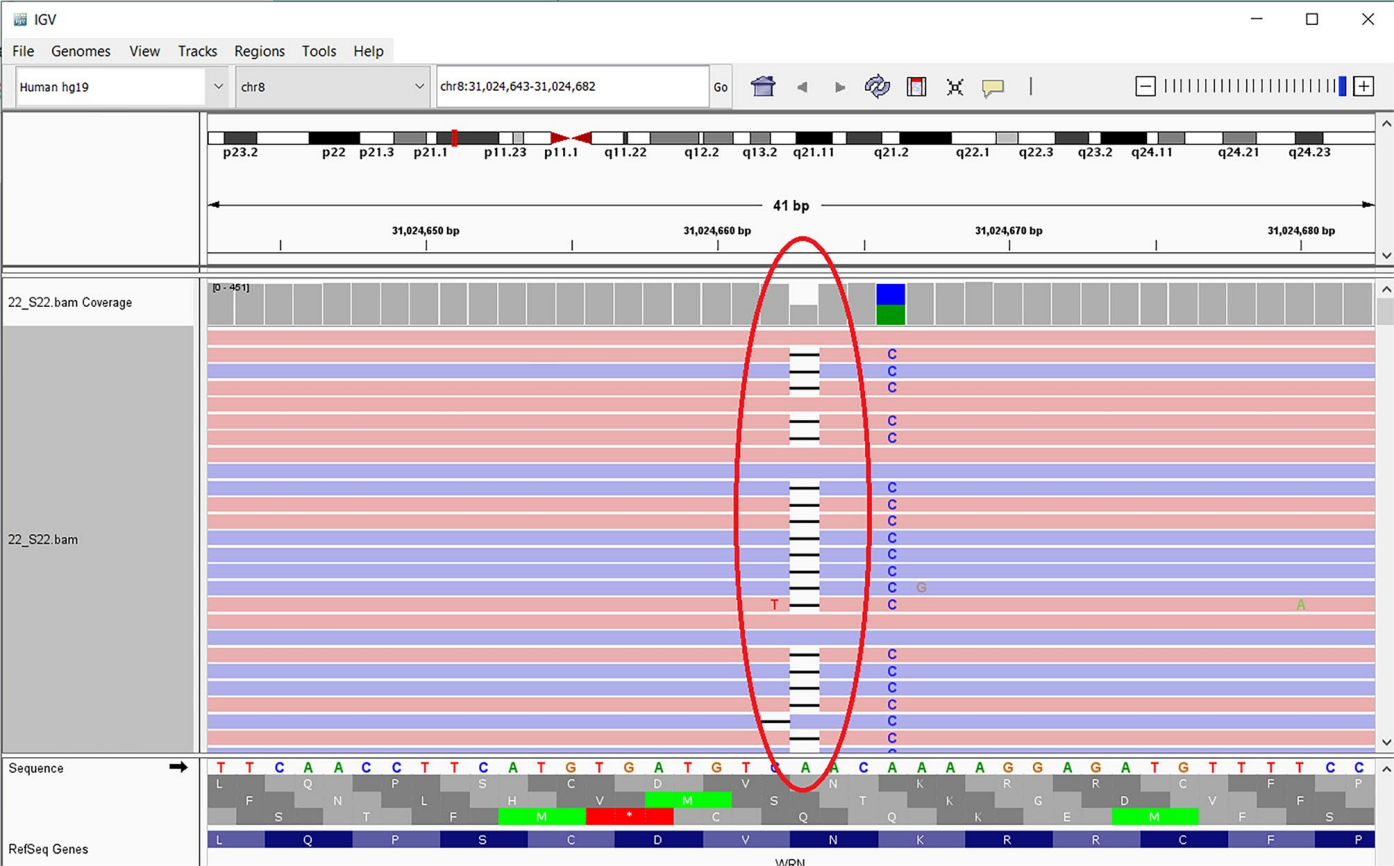
IGV image of the likely pathogenic variant in the WRN gene - c.4109del, p.(Asn1370ThrfsTer23) (NM_000553.4)

Genetic testing (NGS) of the proband revealed a likely pathogenic variant of the WRN gene - c.4109del, p.(Asn1370ThrfsTer23) (NM_000553.4) (Fig. 4).

**Fig. 5:**
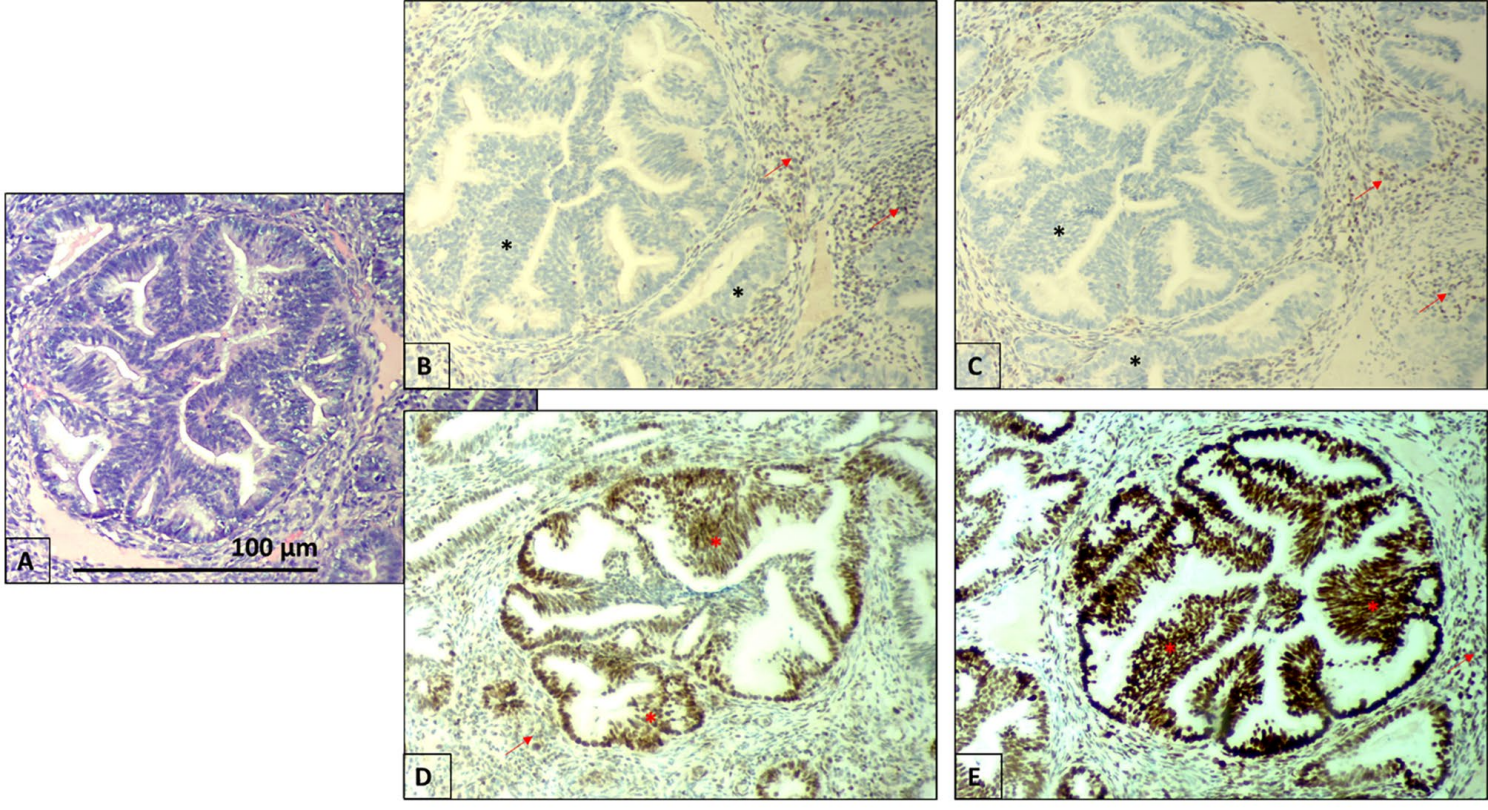
IHC analysis showing the loss of MLH1 **(B)** and PMS2 **(C)** nuclear protein expression (black stars) and preserved MSH2 **(D)** and MSH6 **(E)** nuclear expression (red stars), performed on the endometrial tumor tissue of the proband. Positive internal controls showed preserved nuclear expression in the stromal cells and lymphocytes (red arrow). IHC (Immunohistochemistry), magnification x400. For comparison – **(A)** - hematoxylin and eosin staining of the same slice, magnification x400

To correlate properly the clinical diagnosis with the detected variant in the WRN gene, the genetic counselor recommended IHC testing of endometrial tumor tissue for MMR deficiency. The result was - loss of MLH1 and PMS protein expression (Fig. 5).

Since there are no established guidelines for the management of patients with pathogenic germline variants of the WRN gene, the genetic counselor summarized the information from the clinical, familial, and IHC data and made a recommendation for the patient: a high-quality colonoscopy to be performed and repeated every 3 years, a clinical examination of the breasts every 6 months to 1 year, and a mammogram/MRI of the breasts once a year. The recommendation for the first- degree relative (sister) was to perform a germline test for the familial pathogenic WRN variant.

## Discussion

Endometrial cancer is the most commonly diagnosed gynecologic cancer in women worldwide [27], and approximately 2–12% of patients carry germline mutations in cancer predisposition genes [28–30]. It is usually diagnosed at an early stage, as the first symptom - vaginal bleeding - occurs, and therefore has a good prognosis [31, 32]. The median age at diagnosis is 68 years [33]. Ovarian cancer is less common but is usually diagnosed at late stages. About 10–20% of OC are due to hereditary predisposition [1]. About 5% of endometrial cancers and 10–20% of ovarian cancers are synchronous (the two primary cancers are diagnosed within 6 months) [31, 32]. Synchronous endometrial and ovarian carcinoma (SEOC) accounts for 50–70% of all synchronous gynecologic cancers in women [34]. The typical histology of SEOC is endometroid adenocarcinoma of both the endometrium and ovary, which has been described in up to 70% of cases [1]. The presented clinical case was a postmenopausal 52-year-old woman with histologically verified endometroid adenocarcinoma of the endometrium and synchronous endometroid ovarian cancer. Thus, our case confirms that the majority of SEOC cases have endometroid adenocarcinoma in both the endometrium and ovary.

The presence of SEOC is an independent indication for the evaluation of Lynch syndrome [20]. In our case, there were two other indications for suspicion of hereditary cancer - the endometroid histology of both tumors and the family history of the proband. All these features were the reason for the referral of the presented patient to our genetics department. Because the clinical data indicated LS, we expected to find a pathogenic germline variant in the MMR gene. Instead, the result of the germline gene test was a likely pathogenic variant (c.4109del) in the WRN gene.

The WRN gene encodes the WRN helicase, which belongs to the RecQ family of helicases [35]. In humans, there are five different RecQ helicases - RecQ1, BLM, WRN, RecQ4 and RecQ5. They belong to the family of DNA unwinding enzymes that are essential for maintaining genomic stability by repairing damaged DNA, signaling DNA damage, maintaining telomeres, base excision repair, and homologous recombination. The five helicases have similar domains: the core helicase domain, the RecQ C terminal domain and the helicase and RnaseD-like C-terminal (HRDC) domain [36]. Biallelic inactivation of the WRN gene results in autosomal recessive Werner syndrome (OMIM 277,700), which is characterized by a premature aging phenotype, short stature, early graying, bilateral cataracts, and other features [37]. In vitro experiments confirmed that cell lines in the case of a heterozygous state (monoallelic carrier state of the pathogenic variant) have reduced WRN proteins and helicase activity, which could predispose to cancer [38].

The pathogenic/likely pathogenic variants in the WRN gene reported to date account for 324, one-third of which are frameshift and, less frequently, nonsense and splice-cite mutations [39]. In the present case, we have discovered a likely pathogenic variant - a frameshift mutation that is the result of a deletion of adenine at 4109 site of the WRN nucleotide sequence (1370 aa site in the protein). This variant results in alteration of the WRN protein after 1370 aa and shortening after 23 amino acids. The truncation of the WRN gene leads to a loss of nuclear localization signal (NLS) in the C-terminal region (aa1370-1375) and, in addition, the altered WRN protein could not be transported into the nucleus [40].

The detected variant is not present in the population database (GnomAD) but has been identified in affected individuals in a Chinese family [41]. One of the affected relatives in the Chinese family developed endometrial cancer at the age of 55 years, which is consistent with the clinical data of our case (diagnosed at the age of 52 years). In addition, the truncated variant was confirmed in two other affected females from the Chinese family. In our case, the other affected family members are also female, but they are no longer alive and we could not confirm the carrier status of the WRN variant, but based on their clinical data, we suspect that they were also carriers of the familial WRN variant. One of the affected family members, from our case, was diagnosed with breast cancer, and it is known that pathogenic WRN variants were found quite frequently (2.95%) in breast cancer patients [42]. The other family member was diagnosed with ovarian cancer, which is not as common in WRN mutation carriers but is part of the Lynch syndrome cancer spectrum. Our proband’s sister (healthy, 48 years old) underwent genetic testing at another laboratory and informed the genetic counselor that she was negative for a familial variant in the WRN gene.

In the present clinical case, the detected pathogenic variant is in the heterozygous state and has not been confirmed in unaffected relatives. Since WRN mutations (WRN-mut) in cancer lead to genomic instability and in order to establish the correct correlation between the pathogenic germline variant and the clinical diagnosis, the genetic counselor recommended a histological IHC evaluation of the MMR status of the proband’s tumor tissue. The result showed that the tumor was MMR deficient (no expression of MLH1 and PMS).

All clinical, familial, genetic and histological data of our clinical case lead to the final conclusion that this case is a hereditary LLS.

The cumulative risk of developing cancer at age 70 was found to be higher in LLS than in the general population and lower than in LS. Nevertheless, these patients and their families should be considered at high-risk and eligible for prevention strategies [19]. There are different recommendations for prevention in LLS patients and their first-degree relatives, those that make the recommendation similar to LS with longer surveillance intervals [18] and others that take into account the age for diagnosis of LLS (between the age for the general population and the age at LS) and family history [43]. Considering the family history of the presented clinical case, we gave the proband the compiled recommendation for prevention - a clinical examination of the breasts every 6 months to 1 year and a mammography/MRI of the breasts once a year and because in LLS the most commonly affected organ is the colon - in our case a high quality colonoscopy to be performed and repeated every 3 years.

The presented clinical case could contribute to the identification of the etiology of LLS. Based on the combined information from clinicians, pathologists, genetic counselors, and big data from NGS testing for cancer predispositions, clinical surveillance and follow-up management in women with gynecologic cancers, especially SEOC, could be improved.

## Data Availability

The datasets used and/or analyzed during the present study are available from the corresponding author on reasonable request.

## List of abbreviations

OC: ovarian cancer
SEOC: Synchronous endometrial and ovarian cancer
LS: Lynch syndrome
LLS: Lynch-like syndrome
NGS: next–generation sequencing
MMR: mismatch repair
